# Microglial Implications in SARS-CoV-2 Infection and COVID-19: Lessons From Viral RNA Neurotropism and Possible Relevance to Parkinson’s Disease

**DOI:** 10.3389/fncel.2021.670298

**Published:** 2021-06-15

**Authors:** Ifeoluwa O. Awogbindin, Benneth Ben-Azu, Babatunde A. Olusola, Elizabeth T. Akinluyi, Philip A. Adeniyi, Therese Di Paolo, Marie-Ève Tremblay

**Affiliations:** ^1^Division of Medical Sciences, University of Victoria, Victoria, BC, Canada; ^2^Neuroimmunology Group, Molecular Drug Metabolism and Toxicology Laboratory, Department of Biochemistry, College of Medicine, University of Ibadan, Ibadan, Nigeria; ^3^Neuropharmacology Unit, Department of Pharmacology and Therapeutics, Faculty of Basic Medical Sciences, College of Health Sciences, Delta State University, Abraka, Nigeria; ^4^Department of Virology, College of Medicine, University of Ibadan, Ibadan, Nigeria; ^5^Department of Pharmacology and Therapeutics, College of Medicine and Health Sciences, Afe Babalola University, Ado-Ekiti, Nigeria; ^6^Department of Comparative Biomedical Sciences, School of Veterinary Medicine, Louisiana State University, Baton Rouge, LA, United States; ^7^Axe Neurosciences, Centre de Recherche du CHU de Québec-Université Laval, Québec, QC, Canada; ^8^Faculté de Pharmacie, Université Laval, Québec, QC, Canada; ^9^Neurology and Neurosurgery Department, McGill University, Montréal, QC, Canada; ^10^Department of Molecular Medicine, Université Laval, Québec, QC, Canada; ^11^Department of Biochemistry and Molecular Biology, University of British Columbia, Vancouver, BC, Canada

**Keywords:** microglia, SARS-CoV-2, COVID-19, brain, viral RNA neurotropism, Parkinson’s disease, neuropsychiatric disorders, neurodegenerative diseases

## Abstract

Since December 2019, humankind has been experiencing a ravaging severe acute respiratory syndrome coronavirus 2 (SARS-CoV-2) outbreak, the second coronavirus pandemic in a decade after the Middle East respiratory syndrome coronavirus (MERS-CoV) disease in 2012. Infection with SARS-CoV-2 results in Coronavirus disease 2019 (COVID-19), which is responsible for over 3.1 million deaths worldwide. With the emergence of a second and a third wave of infection across the globe, and the rising record of multiple reinfections and relapses, SARS-CoV-2 infection shows no sign of abating. In addition, it is now evident that SARS-CoV-2 infection presents with neurological symptoms that include early hyposmia, ischemic stroke, meningitis, delirium and falls, even after viral clearance. This may suggest chronic or permanent changes to the neurons, glial cells, and/or brain vasculature in response to SARS-CoV-2 infection or COVID-19. Within the central nervous system (CNS), microglia act as the central housekeepers against altered homeostatic states, including during viral neurotropic infections. In this review, we highlight microglial responses to viral neuroinfections, especially those with a similar genetic composition and route of entry as SARS-CoV-2. As the primary sensor of viral infection in the CNS, we describe the pathogenic and neuroinvasive mechanisms of RNA viruses and SARS-CoV-2 vis-à-vis the microglial means of viral recognition. Responses of microglia which may culminate in viral clearance or immunopathology are also covered. Lastly, we further discuss the implication of SARS-CoV-2 CNS invasion on microglial plasticity and associated long-term neurodegeneration. As such, this review provides insight into some of the mechanisms by which microglia could contribute to the pathophysiology of post-COVID-19 neurological sequelae and disorders, including Parkinson’s disease, which could be pervasive in the coming years given the growing numbers of infected and re-infected individuals globally.

## Introduction

Since the turn of 2019, a widespread and lingering severe acute respiratory syndrome coronavirus 2 (SARS-CoV-2) pandemic has been ravaging the humanity (Rabi et al., 2020). Coronaviruses are large, pleomorphic, enveloped, and positive-stranded RNA viruses that belong to three genera: alphacoronavirus, betacoronavirus, gammacoronavirus. These viruses are responsible for a wide range of respiratory, gastrointestinal, hepatic, and neurological diseases with varying severity levels (Jin et al., 2020; Vargas et al., 2020). SARS-CoV-2 belongs to the genus betacoronaviruses of the family (Walker et al., 2019). Examples of previously existing coronaviruses are severe acute respiratory syndrome coronavirus 1 (SARS-CoV-1) and Middle East respiratory syndrome coronavirus (MERS-CoV), which were the causes of the 2002 and 2012 epidemics, respectively (Vargas et al., 2020). Others are the human coronavirus 229E (HCoV-229E; one of the first alphacoronavirus strains to be reported), human coronavirus OC43 (HCoV-OC43; a betacoronavirus), human coronavirus NL63 (HCoV-NL63; an alphacoronavirus), and human coronavirus HKU1 (HCoV-HKUI; a betacoronavirus) (Fehr and Perlman, 2015; Chen Y. et al., 2020).

The genome of SARS-CoV-2 is 79.5% similar to other SARS-CoVs. This shows extensive sequence homology and conservation within the family (Choudhury and Mukherjee, 2020; Jin et al., 2020; Motayo et al., 2020). The major structural proteins are the spike, matrix, envelope, and nucleocapsid (Jin et al., 2020; Onofrio et al., 2020). However, a significant striking difference between SARS-CoV-2 and other coronaviruses is the longer length of the spike protein amino acid (Onofrio et al., 2020). This disparity has been suggested to confer higher transmissibility potential to SARS-CoV-2, making it possible for the virus to infect humans of different races and geographical origins (Onofrio et al., 2020). As of May 1, 2021, more than 1.9% of the world population are infected with SARS-CoV-2. The infection has been reported in over 86.85% of countries and territories globally with over 3.1% deaths (World Health Organization [WHO], 2021). Infection with SARS-CoV-2 results in Coronavirus disease 2019 (COVID-19) with a signature collection of symptoms including dyspnea, fatigue, pulmonary insufficiency, fever, dry cough, nasal congestion, myalgia, headache, and intestinal dysfunction (Wiersinga et al., 2020).

With a second surge in reported cases globally and growing numbers of multiple re-infections or relapses, SARS-CoV-2 infection shows no sign of abating (Iwasaki, 2020). Notably, re-infection or relapses, characterized by inflammatory rebound, pose more threats than the previous infection and commonly result in fatalities (Gousseff et al., 2020; Lafaie et al., 2020). Homing of SARS-CoV-2 into the cells of vulnerable organs relies on angiotensin-converting enzyme 2 (ACE2) binding (Letko et al., 2020), while neuropilin-1 (NRP-1) has recently been discovered as a receptor capable of facilitating SARS-CoV-2 entry with ACE2-independent mechanism (Cantuti-Castelvetri et al., 2020). SARS-CoV-2 can enter various organs displaying abundant ACE2 and NRP-1 expression, such as the nasopharynx, lungs, stomach, small intestine, lymph nodes, spleen, kidney, and brain (Hamming et al., 2004). The severity of COVID-19 results in complications and loss of function across these multiple organs, especially in individuals with co-morbidities (Zou et al., 2020). Also, SARS-CoV-2 infection results in neurological symptoms, including early hyposmia, reduced or total inability to detect odors, ischemic stroke, meningitis, cerebral thrombosis, delirium, and dizziness (Ahmad and Rathore, 2020). Moreover, documented and non-documented reports of neuropsychiatry symptoms and movement abnormalities post-infection are also emerging (Poyiadji et al., 2020; Thakur et al., 2021). Evidently, some reports have described the lack of coordination in movement, gait shuffling, falls, confusion, and clumsiness of thoughts, even after SARS-CoV-2 antibodies were detected and testing negative to the virus for several days (Mao et al., 2020; Piscitelli et al., 2020). This may suggest a continuous or permanent remodeling of ACE2-expressing neurons, glial cells including microglia, and/or brain vasculature in response to SARS-CoV-2 or COVID-19. However, very few pathophysiological details are currently available.

In this review, we highlight microglial response to viral neuroinfections, especially those with similar genetic composition and route of entry as SARS-CoV-2, given that microglia have been implicated in the pathophysiology of viral-mediated neurodevelopmental, neuropsychiatric, and neurodegenerative disease conditions (Rock et al., 2004). Microglia are the primary non-neuronal innate sensors of viral infections in the central nervous system (CNS) (Chen et al., 2019). They survey the CNS parenchyma, respond to viral stressors, and regulate the egress of viral particles across brain regions (Nimmerjahn et al., 2005; Fekete et al., 2018). Specifically, we describe the available literature on SARS-CoV-2 neurotropism, with a special focus on its route of entry to the CNS. Furthermore, we discuss a few neurotropic RNA viruses with similar route of entry to the CNS, providing specific details on the interaction between viral pathogen-associated molecular patterns (PAMPs) and microglia-expressed receptors. Since microglia are important mediators of neuroprotection and neurodegeneration in the CNS (Chen and Trapp, 2016; Tay et al., 2017; Bellver-Landete et al., 2019), we emphasize the outcomes of SARS-CoV-2 CNS invasion on microglial responses. Further, we provide concise explanations on the specific microglial response vis-à-vis the associated immediate and long-term implications. Consequently, this article offers insight into possible pathogenic mechanisms by which microglial reactivity during SAR-CoV-2 infection could be involved in the development of post-COVID-19 neurological sequelae and syndromes, including Parkinson’s disease.

## Evidence of SARS-CoV-2 Neurotropism

Increasing number of reports describe a prevalence of neurological alterations and symptoms in COVID-19 (Brown et al., 2020; Helms et al., 2020; Mao et al., 2020; Piscitelli et al., 2020). Accordingly, the neurotropic hypothesis and capacity of SARS-CoV-2 were first proposed following the manifestation of anosmia in COVID-19 patients, as observed in other coronaviruses (Su et al., 2016; Dubé et al., 2018; Qian et al., 2020). Among the common neuropsychiatric presentations, delirium is suggested to be the most prevalent in over 30% of patient during the acute phase of SARS-CoV-2 infection (Rogers et al., 2020). Although delirium is often believed to occur as an early prodromal of mild brain dysfunction, in the case of infectious diseases, delirium can be caused by aggravated peripheral inflammatory response or be a direct effect of infectious agents on the CNS (Tsuruta and Oda, 2016).

In line with the neuropathological sequelae, mined data from brain single-nuclear RNA sequencing and spatial distribution analyses found relatively high levels of ACE2 expression in several brain regions including the choroid plexus, *substantia nigra* (SN), ventricles, and neurovasculature (Chen T. et al., 2020; Hu X. et al., 2020). Results from the analysis of single-cell sequencing data from human middle temporal gyrus also found that ACE2 was widely distributed in human and mouse neurons, astrocytes and oligodendrocytes, but rarely in microglia (Chen R. et al., 2020). An ultrastructural characterization of the spike protein component of the virus by high-resolution cryoelectron microscopy further revealed that ACE2 was critical for the neuroinvasiveness of SARS-CoV-2 (Wrapp et al., 2019). In comparison with previous coronaviruses, the study also reported that the spike protein octodomain of SARS-CoV-2 had a higher affinity for the ACE2 receptor (Wrapp et al., 2019), suggesting that SARS-CoV-2 may have a more potent neuroinvasive property (Natoli et al., 2020).

In some instances, specific SARS-CoV-2 antibodies and viral antigens have been confirmed in the cerebrospinal fluid (CSF) of infected individuals by genomic sequencing (Benameur et al., 2020; Moriguchi et al., 2020; Wu et al., 2020). Radiographic examinations showed traces of COVID-19-associated neuropathological features, including acute hemorrhagic necrotizing encephalitis (AHLE) and acute disseminated encephalomyelitis (ADEM) (Poyiadji et al., 2020; Reichard et al., 2020; Thakur et al., 2021). A biopsy examination has been used to confirm temporal lobe encephalitis enriched by perivascular lymphocytic infiltration, microglial nodule, and neuronal destabilization (Hu J. et al., 2020; Thakur et al., 2021). As such, it has been hypothesized that the auto-antigenicity of SARS-CoV-2 antibodies causes macrophage functional recruitment and ADEM. The immunological reactivity of SARS-CoV-2 is crossed-linked to human neuronal myelin sheath and thus may likely promote a post-infectious autoimmune demyelinated pathology of the brain (Garg et al., 2020; Parsons et al., 2020). Furthermore, evidence derived from forebrain specific human neural progenitor cells (hNPCs) generated from human-induced pluripotent stem cell (hiPSC) lines demonstrated replication of SARS-CoV-2 in 2-week old hNPCs with peak infection in less than 12 h post-infection. The study showed increased cell death with TUNEL assay (Song et al., 2020). The hiPSC-derived brain organoids showed at 9 weeks increased viral particles and infection of microtubule-associated protein 2 (MAP2)-positive mature neurons 24 h post-infection. The study suggested that SARS-CoV-2 can infect cells of neural origin and hypothesized that infected cells can cause death of nearby cells (Solomon et al., 2020). In comparison with other viruses, such as Zika virus (ZIKV), SARS-CoV-2 related brain infection has been linked to an upregulation of cell division, organelle fission, and metabolic processes, via moderate interferon-stimulated gene activation (Blanco-Melo et al., 2020). This finding suggest that the brain is a site of high replicative potential for SARS-CoV-2 (Song et al., 2020). Additionally, using transgenic mice expressing ACE2 under the K18 promoter (hACE2-K18), intranasal administration of SARS-CoV-2 (Song et al., 2020) resulted in viral titers in the brain, especially in the forebrain and cerebral cortex. However, low distribution in the dentate gyrus, globus pallidus, and cortical layer 4 was reported. Together, these findings suggest that SARS-CoV-2 can infect patients brain’s neurons via its neurotropic properties (Song et al., 2020). However, given that microglia act as the central housekeepers against altered homeostatic states, even during viral neurotropic infections, it is tempting to speculate that microglial reactivity and cell infectivity by SARS-CoV-2 might play an important role in the transmission and activity of the virus across the brain parenchyma (Vargas et al., 2020).

## Route of Entry and Neuroinvasive Mechanisms of SARS-CoV-2 and RNA Viruses

Neurotropic RNA viruses gain access to the CNS via different routes. While some viruses overcome the barrier between the periphery and CNS, a few usurp the direct access between the olfactory canal and the brain (Chen G. et al., 2020; Hu X. et al., 2020). Other viruses channel the retrograde transport between peripheral and central nervous systems before achieving neuroinfection (Chen G. et al., 2020; Hu X. et al., 2020). For instance, West Nile virus (WNV), Canine distemper virus, and Mumps virus breach the blood-brain barrier (BBB), meningeal blood-barrier, and blood-CSF barrier to invade the brain after achieving a significant viremia (Rudd et al., 2006). Using this hematogenous approach, these viruses infect the CNS vasculature endothelium or “trojan horse” lymphocytes, which extravasate through the structural layers of the barrier, before spreading to the adjoining glial cells and neurons within the brain parenchyma. In the process, the virus can infect the components of the protective barriers, including pericytes, astrocytes, microglia, and endothelial cells, thereby increasing their permeability (Miner and Diamond, 2016). Various immune cells derived from the periphery, such as circulating macrophages and dendritic cells, may then crosstalk with the CNS’s neuronal and non-neuronal components (Aghagoli et al., 2020). Following brain invasion, viral proteins can act as PAMPs or induce damage-associated molecular patterns (DAMPs), which in turn trigger an innate immune response via pattern recognition receptors (PRRs) (Dantzer, 2018). Alternatively, airborne coronaviruses in the upper respiratory tract mucosa can infect the bipolar olfactory sensory neurons with unmyelinated axons that penetrate the cribriform plate into the olfactory bulb and form a direct synapse with the mitral cells (Aghagoli et al., 2020; Hu X. et al., 2020). As such, the virus is transmitted transneuronally by usurping the neuronal dynein-kinesins transportation network via anterograde axonal transport within the olfactory receptor neurons and via synaptic transfers to the olfactory bulb mitral cells. Following the linear anterograde and retrograde transport between the olfactory bulb and different bran regions, the virus can access the limbic system and other deeper nuclei (Aghagoli et al., 2020; Hu X. et al., 2020).

There are three primary routes of SARS-CoV-2 transmission: droplets, aerosol, and contact (Adhikari et al., 2020; Kabir et al., 2020; Qian et al., 2020; Zhang et al., 2020a). Cell and tissue tropism determines the host restriction (Lee et al., 2020; Saxena et al., 2020). Similar to SARS-CoV-1, SARS-CoV-2 spike protein binds to ACE2, even with a 10–20-fold higher affinity (Jin et al., 2020). However, the fusion requires a proteolytic activation by transmembrane protease serine 2 (TMPRSS2) as well as cathepsin B and L for full entry (Letko et al., 2020; Wan et al., 2020). The ubiquitous nature of ACE2 enables SARS-CoV-2 to gain access to various cell types and elicit multiorgan failure. It is proposed that SARS-CoV-2 neuroinvasion is possible essentially through the hematogenous and transneuronal routes (Chen G. et al., 2020; Hu X. et al., 2020). For instance, autopsied brain analysis by electron microscopy of COVID-19 patients showed endotheliitis, mononuclear inflammatory cells, and viral-like structures in the brain capillary endothelium of the frontal lobe (Chigr et al., 2020; Varga et al., 2020). This is suggestive of direct endothelial viral infection. Furthermore, because ACE2 is expressed in both the lymph node-associated CD68-positive (^+^) macrophages and tissue-based CD169^+^ macrophage, the trojan horse mechanism of SARS-CoV-2 neuroinvasion may involve a number of mechanisms. First, a viral infection of leukocytes or monocyte-macrophages before a paracellular and circumventricular transfer to the brain, mainly through organs that are deficient in BBB (Chen G. et al., 2020). It can also proceed via dorsal root or autonomic ganglia. A detailed mechanistic study in mice revealed that S1 subunit of SARS-CoV-2 spike protein was initially retained on the luminal side of capillary bed after which it crossed the BBB via adsorptive transcytosis with an intense tropism in different brain regions including the striatum, midbrain, hypothalamus and olfactory bulb, following intravenous injection (Rhea et al., 2020). This suggests that the S1 subunit may drive the brain uptake of SARS-CoV-2 through the BBB. Shed SARS-CoV-2 S1 subunit may also display the full pathogenic attributes of whole SARS-CoV-2, even after viral clearance, since it is very stable in the brain and can bind to ACE2 (Rhea et al., 2020). That anosmia and hypogeusia are early neurological alterations mediated by SARS-CoV-2 suggests CNS invasion through the olfactory system. In hACE2-K18 mice, SARS-CoV-1 infects the brain majorly through the olfactory nerve and subsequently spreads to other brain regions transneuronally (Netland et al., 2008). Similarly, in patients with COVID-19, recent images from fluid-attenuated inversion recovery (FLAIR) on magnetic resonance imaging (MRI) sequence revealed a bilateral hyper-intensity of the olfactory bulbs and right gyrus rectus (Politi et al., 2020). This further reinforces the transneuronal or olfactory pathway hypothesis (Hu J. et al., 2020). This finding could, in part, provide a possible mechanism underlying the cranial nerve symptoms observed in COVID-19.

Some studies have also highlighted the possibility of SARS-CoV-2 transmission through the digestive tract and the vagus nerve (Adhikari et al., 2020; Kabir et al., 2020; Qian et al., 2020; Zhang et al., 2020a). Previous findings showed that nucleus tractus solitarius and dorsal motor nucleus of the vagus nerve, which connect and activate the central autonomic network (sympathetic and parasympathetic systems) (Benarroch, 1993), express ACE2 (Doobay et al., 2007). Thus, SAR-CoV-2 is capable of infecting the terminal structures of the vagal afferents and the early parts of the vagal efferents to cause downexpression of ACE2, a mechanism that is already being implicated in disease severity and organ damage (Bonaz et al., 2020). Emerging evidence indicates that dysregulated cholinergic anti-inflammatory cascade by SAR-CoV-2 is linked to leaky gut, immune reactivity, BBB breakdown, and microglial reactivity (Changeux et al., 2020). Vagus nerve stimulation profoundly attenuated the microglial reactivity in lipopolysaccharide (LPS)-exposed mice (Meneses et al., 2016) and SARS-CoV-2-induced symptoms in COVID-19 patients (Changeux et al., 2020). Although it remains a hypothesis, two possible mechanisms may be used by SARS-CoV-2 to infect intestinal cells. The first has been described previously to occur during acute entero- and retroviral infections of the intestine. This process includes the disruption of the gut microbiota by these invading organisms, leading to the release of plasma LPS and other inflammatory biomarkers. The systemic inflammation leads to dysbiosis together with water and electrolyte imbalances causing gastrointestinal symptoms (Bergamini et al., 2018; Rao, 2020). The other mechanism has been reviewed elsewhere (de Oliveira et al., 2020). This hypothesis involves the downregulation of ACE2 on intestinal cells after SARS-CoV-2 binding. Reduction in the expression of ACE2 leads to decreased activation of the mechanistic target of rapamycin (mTOR) signaling cascade, which signals exacerbated inflammation, leaky gut, and increased autophagy (Chiappini et al., 2020; da Silva et al., 2020).

## Viral PAMPs and DAMPs Following RNA Virus CNS Infection

RNA viruses, which are mostly old-world viruses (e.g., measles, mumps, rubella, rabies) or zoonotic, i.e., transmitted from other animals to humans, either through insect vectors (arthropod-borne, e.g., West Nile and flaviviruses), rodents (rodent-borne, e.g., Lassa fever viruses) or originating from bats (Lyssaviruses, SARS-CoV-2), are highly neuroinvasive and associated with CNS infections (Lund et al., 2004; Wang et al., 2006; Cui et al., 2010; Faul et al., 2010; Furr and Marriott, 2012; Jureka et al., 2015; Sallenave and Guillot, 2020). Viral CNS infections result in exacerbated inflammation responsible for several symptoms, such as fever, headache, confusion, stroke, seizure, or death (Furr and Marriott, 2012). Such infections trigger reactivity of resident immune and glial cells that serve as defenses for the CNS, namely, microglia and astrocytes. This results in the release of proinflammatory cytokines and subsequent activation of both innate and adaptive immune responses. These immune responses may limit the viral replication and spread; however, sustained inflammation can elicit damage to the function and structure of neurons directly or indirectly (Ising and Heneka, 2018). For example, early induction and secretion of interferons may protect neighboring cells from viral infection (see section “Microglial Response During RNA Viral Infections”). However, inflammatory mediators can also impact negatively on neuronal function at synapses thus affecting interneuronal networks. For instance, under an inflammatory environment, microglia may lose their homeostatic function of synaptic pruning and remodeling, notably due to process retraction (Stence et al., 2001; Schafer et al., 2012). Glial-mediated trophic support secretion also reduces under inflammatory conditions, resulting in functional and structural impairment of neurons (Parkhurst et al., 2013; Pöyhönen et al., 2019). Evidence also revealed that suppression of synaptic plasticity particularly long-term potentiation co-exists with sustained levels of tumor necrosis factor (TNF)-α (Tancredi et al., 1992), interleukin (IL)-6 (Tancredi et al., 2000), nitric oxide synthase 2 (Wang et al., 2004), and complement factor 3 (C3) (Lian et al., 2015) in the brain parenchyma. These inflammatory mediators may modulate astrocytes to trigger neurodegeneration (Liddelow et al., 2017).

Viral epitopes are recognized by pathogen recognition receptors (PRRs) or sensors on the plasma membrane, in the endosome, and within the immune cells’ cytoplasm. These PRRs interact with conserved PAMPs and nucleic acids, as well as DAMPs (Pichlmair and Reis e Sousa, 2007; Pedraza et al., 2010). The PAMPs are unique footprints of pathogens that are conserved among similar pathogens. While membrane-bound and endosomal PRRs, which target viral single-stranded (ss) and double-stranded (ds) RNA and DNA, sufficiently discriminate between self and exogenous nucleic acids, the cytoplasmic sensors, particularly those that bind to dsDNA, do not differentiate these nucleic acids (Roers et al., 2016). The PRRs (Table 1) include Toll-like receptors (TLRs), nucleotide oligomerization domain (NOD)-like receptors (NLRs), and retinoic acid-inducible gene (RIG)-I-like receptors (RLRs), and cytosolic DNA sensors, such as cyclic GMP-AMP synthase (cGAS) (Pedraza et al., 2010; Jensen and Thomsen, 2012; Said et al., 2018; Lee et al., 2019).

**TABLE 1:** Pattern recognition receptors (PRRs) with presence in microglia: recognized viruses and ligands.

Receptor	Virus	Ligand	References
TLR2	Measles virus, Hepatitis C virus	HA; Core protein; NS3	Bieback et al., 2002; Pedraza et al., 2010
TLR3	Respiratory syncytial virus, West Nile virus, Influenza A virus, Coxsackievirus B3, Polio virus	dsRNA; Poly (I:C)	Guillot et al., 2005; Kato et al., 2005; Wang et al., 2006; Daffis et al., 2008; Negishi et al., 2008; Oshiumi et al., 2011
TLR4	Respiratory syncytial virus, Mouse mammary tumor virus	Fusion protein; Envelope protein	Zhou et al., 2010
TLR7	Influenza A virus, Vesicular stomatitis virus, Human Immunodeficiency virus, Dengue virus, Respiratory syncytial virus, Coxsackievirus, Ebola, Yellow fever virus, Poliovirus, Rhinovirus, Human T-lymphotropic virus type I/II	ssRNA	Lund et al., 2004; Beignon et al., 2005; Wang et al., 2006; Hardy et al., 2007
TLR8	Human Immunodeficiency virus, Respiratory syncytial virus, Coxsackievirus, Influenza A virus, Hepatitis C virus, Rhinoviruses, Yellow fever virus	ssRNA	Jensen and Thomsen, 2012
RIG-I	Respiratory syncytial virus, Measles virus, Nipah virus, Rabies virus, Influenza A virus, Ebola virus, Lassa fever virus, Lymphocytic choriomeningitis virus, Japanese encephalitis virus, Hepatitis C virus, West Nile virus, Dengue virus	ss/dsRNA	Kato et al., 2005; Cárdenas et al., 2006; Plumet et al., 2007; Fredericksen et al., 2008; Habjan et al., 2008; Loo et al., 2008; Faul et al., 2010; Zhou et al., 2010
NOD NLRP3	Influenza A virus	Virus- cell stress	Kanneganti et al., 2006
NLRC2	Respiratory syncytial virus, Influenza A virus, parainfluenza virus	ssRNA	Jensen and Thomsen, 2012
MDA5	Encephalomyocarditis virus, Rabies virus, West Nile virus, Dengue virus, Polio, Coxsackievirus, Rabies virus	dsRNA	Kato et al., 2005; Gitlin et al., 2006; Loo et al., 2008; Faul et al., 2010

Despite their CNS homeostasis and neuronal cell functions, microglia and astrocytes are mainly involved in the protective immune responses during neuroinflammation (Facci et al., 2018; Jha et al., 2019; Tremblay et al., 2020). Following the recognition of PAMPs by PRR, glial cells express antiviral inflammatory mediators including type-1 interferons (IFN), IL-1β, TNF-α, and IL-6, which can induce peripheral immune cell infiltration across the BBB (Chauhan et al., 2008; Furr et al., 2010; Furr and Marriott, 2012; Jensen and Thomsen, 2012; Chen et al., 2017; Lee et al., 2019; Choudhury and Mukherjee, 2020). The endogenous DAMPs produced by stressed or dying neurons can also initiate PRR-mediated inflammatory responses (Nan et al., 2014; Fleshner and Crane, 2017; Lee et al., 2019). Although secreted cytokines are essential for inducing efficient and robust adaptive immune responses, excessive IFN production, as well as prolonged inflammatory responses, together elicit CNS damage, as discussed in the section “Microglial Response During RNA Viral Infections” (Bastard et al., 2020; Zhang et al., 2020d). On the other hand, the ineffective PRR signaling can increase viral infection severity (Lee et al., 2019). Hence, there is the need for a tight regulation of PRRs mediated signal transduction. Viruses have evolved several strategies (Table 2) to evade host immune detection and clearance. These strategies include interruption of viral sensors and manipulating molecules within signaling cascades (Gack et al., 2007; Jensen and Thomsen, 2012; Lee et al., 2019). A myriad of viral proteins target RIG-I because it is pivotal to the signaling cascades leading to the induction and secretion of antiviral IFN. These viral proteins interfere directly with RIG-I. For instance, the nucleocapsid of SARS-CoVs down-regulates RIG-I activity by targeting tripartite motif-containing protein 25 (TRIM25) thereby preventing RIG-I ubiquitination and subsequent IFN production (Pedraza et al., 2010; Furr and Marriott, 2012; Jensen and Thomsen, 2012; Lee et al., 2019).

**TABLE 2:** Viral evasion mechanism for neurotropic RNA virus PRRs.

Pattern recognition receptor	Virus	Virulence factor	Function	References
RIG-I	Influenza A virus	NS1	TRIM25 inhibition	Lifland et al., 2012
	Picornaviruses	3C protein	Cleavage and inhibition	Barral et al., 2009; Xiao et al., 2020
	Middle East respiratory syndrome coronavirus	4A	PACT suppression	Siu et al., 2014
	Severe acute respiratory syndrome coronavirus	N	TRIM25 inhibition	Jureka et al., 2015
	Dengue virus	sfRNA	TRIM25 inhibition	Manokaran et al., 2015
		NS3	Translocation	Chan and Gack, 2016a
	West Nile virus	NS3	Translocation	Chan and Gack, 2016a
		NS1	Proteasomal degradation	Chiang et al., 2017

## Pathogenesis of SARS-CoV-2 Infection

Following entry, there is an incubation period of about 5 days before symptoms begin to appear. It takes around 6–40 days from symptoms appearance to death, although the mean interval is 14 days (Kabir et al., 2020), whereas the mean interval is about 11.5 days for patients over 70 years of age (Jin et al., 2020). Several studies have noted that due to the acute nature of SARS-CoV-2 infection, innate immune responses may play a critical role in determining its eventual outcomes since the rapidity rather than memory of immune responses is important. This is particularly essential in novel viral infections, such as SARS-CoV-2 in which a pre-existing immunity is not possible (Pichlmair and Reis e Sousa, 2007; Ayegbusi et al., 2018; Costela-Ruiz et al., 2020). The acute nature of the infection may also account for the possibility of severe re-infection due to its deceptive imprinting (Kohler and Nara, 2020; Westerhuis et al., 2020). Deceptive imprinting refers to immune evasion mechanisms by certain viruses in which antibodies are mounted against immunodominant epitopes, thereby inducing strain-specific immunity, which offers little or no neutralization activity between serotypes and subtypes. This phenomenon has been described for Human Immunodeficiency virus (HIV), Influenza, and Dengue viruses (Tobin et al., 2008). Since coronaviruses are ubiquitous and certain strains have been identified in common seasonal colds; deceptive imprinting may account for severe COVID-19 cases with higher antibody titers against seasonal coronaviruses compared to SARS-CoV-2 (Westerhuis et al., 2020).

TLRs and RIG-1 are likely involved in viral clearance as well as in the development of severe COVID-19 disease (Jensen and Thomsen, 2012; Chen et al., 2017; Said et al., 2018). Upon recognition of dsRNA motifs, TLR3 recruits TIR-domain-containing adapter-inducing interferon-β (TRIF) adaptor proteins, which results in NF-κB signaling (Jensen and Thomsen, 2012). TLR4 is expressed although at low basal levels in bronchial, epithelial, and alveolar cells. Its expression and activation are increased upon cellular infiltration in response to viral infection (Pedraza et al., 2010; Jensen and Thomsen, 2012; Said et al., 2018). Both myeloid differentiation primary response 88 (MyD88) and TRIF sorting adaptors have been implicated in the proliferation of acute respiratory distress syndrome caused by other respiratory viruses (Jensen and Thomsen, 2012; Totura et al., 2015). Studies in mice have shown that TLR3^–/–^, TLR4^–/–^, and TRAM^–/–^ mice are more susceptible to SARS-CoV infection, although they experience only transient weight loss with no mortality. In contrast, mice deficient in TLR4 adaptor proteins are highly susceptible to SARS-CoV, showing remarkable weight loss, mortality, impaired lung function, and lung pathology. These mice also show acute respiratory distress syndromes and deranged proinflammatory cytokines as well as interferon-stimulated gene signaling (Totura et al., 2015).

*In silico* studies have suggested a strong binding affinity between the spike protein and TLR4 (Choudhury and Mukherjee, 2020). Experimental mouse models of acute respiratory distress induced by multiple causes, including SARS-CoV-1, also showed the protective role of TLR4 (Totura et al., 2015). TLR activation via MyD88 and TRIF dependent pathways is involved in the pathogenesis of SARS-COV-1 (Jensen and Thomsen, 2012). These pathways lead to the production of proinflammatory cytokines (IL-1, IL-6, TNF-α) and type I IFN-α/β. Although necessary for viral clearance, unabated induction of these pathways may lead to a marked increase of a myriad of pro-inflammatory mediators up to a life-threatening level (cytokine storm), neuroinflammation, and autoimmunity, which contribute significantly to immunopathology (Hosseini et al., 2020; Varatharaj et al., 2020; Zhao H. et al., 2020). Autoimmunity may be another key mechanism of SARS-CoV-2 pathogenesis. Autoimmune responses can be induced by molecular mimicry or genetic defects (Jensen and Thomsen, 2012). Production of antiphospholipid autoantibodies may cause coagulopathy and cerebral infarction, which has been reported in patients with severe COVID-19 (Zhang et al., 2020e; Thakur et al., 2021). Studies have also documented an increased cytokine storm susceptibility in individuals with autoimmune diseases (Schwartz and Deczkowska, 2016). The antiviral role of interferons in the clearance of SARS-CoV-1 infection has been previously described (Jensen and Thomsen, 2012). Recent studies have also reported the enrichment in type I and III IFN genes (Bastard et al., 2020) as well as neutralizing autoantibodies against type I IFN-α2 and IFN-ω in patients with severe COVID-19 pneumonia (Zhang et al., 2020d).

The entry of SARS-CoV-2 into susceptible host cells eventually leads to apoptosis, pyroptosis, ACE2 downregulation, and shedding (Yang, 2020; Zhao Y. et al., 2020; Zhou et al., 2020). This, in turn, leads to primary inflammatory responses involving cytokine and chemokine release, antiviral factors expression, pulmonary cell infiltration, vascular permeability, lymphopenia, and acute respiratory distress (Nimmerjahn and Ravetch, 2007; Wang et al., 2008; Haslwanter et al., 2017). The secondary inflammatory responses involve virus-neutralizing antibody complex, which leads to Fc Receptor (FcR) and complement system activation, accompanied by antibody-mediated cellular cytotoxicity (Nimmerjahn and Ravetch, 2007; Wang et al., 2008; Haslwanter et al., 2017). These inflammatory responses lead to skewing of macrophage responses, abrogation of wound healing, monocyte chemoattractant protein 1 (MCP-1) and IL-8 production, acute lung injury, and cellular damage (Liu et al., 2019; Zhang et al., 2020a; Zhao Y. et al., 2020). Uncontrolled pulmonary inflammation and infiltration are accordingly the leading causes of death among SARS-CoV-2 infected individuals (Wang et al., 2008; Liu et al., 2019; Yang, 2020).

## Microglia, the CNS-Resident Innate Immune Cells, as Sensors of CNS Viral Infection

Upon CNS viral infection, inflammatory responses have long been proposed to involve infiltrating peripheral monocytes and leukocytes, as a vast majority of resident CNS cells assumedly lacked immune functions (Sochocka et al., 2017). However, glial cells, most notably astrocytes and microglia, are now recognized as key players in protective and detrimental host responses during CNS disease states (Serramía et al., 2015; Li and Barres, 2018). Microglia are CNS-resident mononuclear phagocytes characterized by a distinctive ramified structure and specific gene expression (Hickman et al., 2013). These cells constitute 5–10% of total brain cells and are derived from embryonic yolk sac precursors (Ginhoux et al., 2010; Gomez Perdiguero et al., 2015), which seed the brain early in development (Gomez Perdiguero et al., 2015). While microglia play a vital role in the maintenance of CNS homeostasis, they are additionally known to dynamically scan the brain parenchyma, detecting the occurrence of pathologies (Schafer et al., 2012; Neniskyte and Gross, 2017). They also contribute to numerous developmental events and physiological processes, such as neurogenesis (Cunningham et al., 2013; Tremblay et al., 2015), programmed cell death (Wakselman et al., 2008), myelination (Voet et al., 2019; Sariol et al., 2020), synaptic remodeling and maturation (Paolicelli et al., 2011; Schafer et al., 2012; Tremblay et al., 2015). As myeloid cells, microglia are immunologically competent, swiftly responding to pathogenic infections within the CNS by modifying their function with a broad spectrum of reactivity states (Aguzzi et al., 2013; Ransohoff, 2016; Stratoulias et al., 2019). Accordingly, it is expected that microglial reactivity and dysfunction (i.e., altered physiological functions) are implicated in practically all CNS infections (Colonna and Butovsky, 2017; Tay et al., 2017; Wolf et al., 2017). Environmental factors including viral infections modulate microglial functions resulting in pathological synaptic remodeling, which culminates in altered cognition and behavior (Shi et al., 2003; Brown, 2012). Indeed, microglial reactivity outlasts the initial immune response with longlasting effects. This reactivity is often characterized of hypercytokinemia, altered ramification and dystrophy, leading to the formation of lysosomal inclusion proteins, up-regulation of proinflammatory cytokine genes, changes in brain neurochemistry and decreased neurogenesis, which can together result in microglial senescence, dystrophy or dysfunction within the CNS microenviroment (Colonna and Butovsky, 2017; Tay et al., 2017).

**FIGURE 1:**
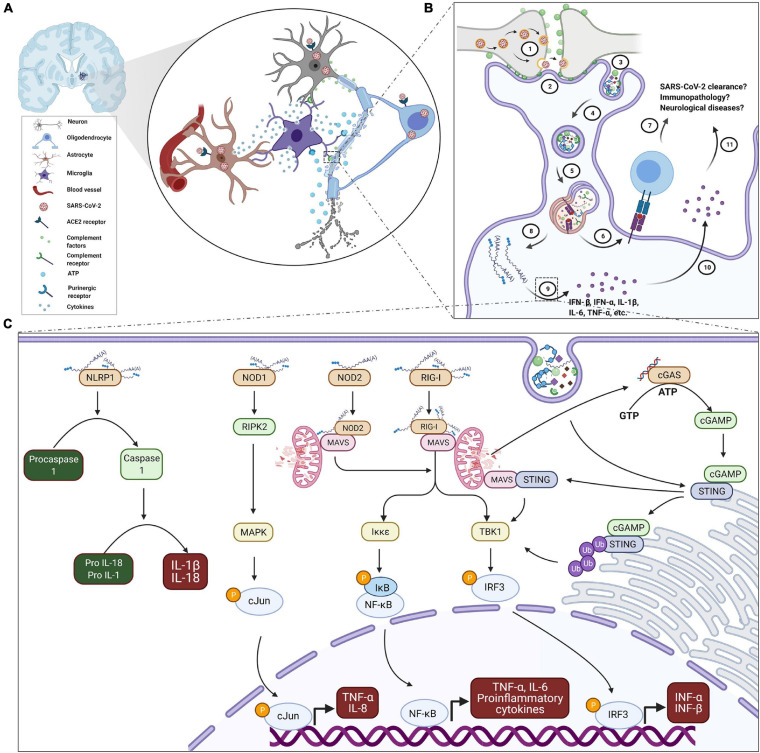
Proposed schematic of microglial reactivity and implications in SARS-CoV-2 infection and COVID-19. **(A)** COVID-19-associated focal hemorrhagic infarcts in the brain are characterized with microglia nodules, degenerating neurons and infiltrated T cells. Thus, microglia may be coordinating the inflammatory events around the infarct’s milieu in a number of ways via reactivity to signals from oligodendrocytes, neurons and astrocytes after SARS-CoV-2 infection, including ATP and complement (C1q or C3) tags, as well as secretion of cytokines. **(B)** For instance, complement coating of SARS-CoV-2-infected synapses (1) may trigger microglial recruitment and interaction via their complement receptors (2) culminating in encapsulation (3) and phagocytosis (4) of synaptic elements in membrane cargoes, which subsequently fuse with lysosomes for adequate processing (5). In the process, fragments of viral peptides may be presented via MHC-I and/or MHC-II to cytotoxic and/or helper T cells (6), respectively, of elicit adaptive immune response. However excessive phagocytosis of synaptic elements may overwhelm the phagolysosomal processing (8) resulting in the exposure of microglia to SARS-CoV-2 genome and functional/structural impairment of vital organelles. The exposure sensitizes microglia to produce (9) and secrete (10) both antiviral and inflammatory cytokines in significant quantity. Although microglia are equipped with a competent innate recognition system, their contribution in the context of SARS-CoV-2 infection and COVID-19 is yet unknown (7 and 11). **(C)** For emphasis, upon cytosolic exposure, microglia may detect SARS-CoV-2 genome through a battery of sensors. NLRP1 sensing of dsRNA and ssRNA activates inflammasome, which processes IL-1β and IL-18 production through caspase 1. NOD1 binding of dsRNA activates the translocation of cJun to the nucleus with subsequent upregulation of pro-inflammatory mediators. RIG-I-bound dsRNA and ssRNA as well as NOD-2- ssRNA complex exacerbate production of TNF-α, IL-6, and IL8 through mitochondrial adaptor protein MAVS mediated NF-κB signaling. Simultaneously, they also regulate the transcription of antiviral type 1 interferons through IRF3. In addition, DAMPs from stressed microglial organelles, such as mitochondrial DNA may trigger cGAS receptor to synthesize cGAMP, an agonist of STING. STING activation potentiates IRF3 signaling. Membrane fusion of endosomatic cargoes may also initiate cGAS-independent STING-interferons signaling through MAVS. Thus, characterization of the specific contribution of microglia in the development of neuronal damage and associated neurological sequelae, or the involvement in debris clearance, SARS-CoV-2 resolution and disease outcome is an active area of research. ATP, adenosine triphosphate; COVID-19, coronavirus disease 2019; DAMPs, damage-associated molecular patterns; cGAMP, cyclic GMP-AMP; cGAS, cyclic GMP-AMP synthase; GTP, guanosine triphosphate; IL, interleukin; IκB, inhibitor of κB; Iκκε, IκB kinase; IFN-α/β, interferon alpha/beta; IRF3, interferon Regulatory Factor 3; MAPK, mitogen activated protein kinase; MAVS, mitochondrial antiviral signaling protein; MHC-I/II, major histocompatibility complex I/II; NOD2/1, nucleotide-binding oligomerization domain 2/1; NF-κB, nuclear factor kappa light chain enhancer of activated B cells; NLRP1, NLR family pyrin domain containing 1; P, phosphate; RIG-I, retinoic acid-inducible gene I; RIPK2, receptor interacting serine/threonine protein kinase 2; dsRNA, double stranded viral RNA; ssRNA, single stranded viral RNA; SARS-CoV-2, severe acute respiratory syndrome coronavirus; STING, stimulator of type I interferon genes; TBK-1, TANK-binding kinase 1; TNF-α, tumor necrosis factor alpha; Ub, ubiquitin.

Microglia are equipped with the viral DAMPs recognition system (Jeffries and Marriott, 2017), which activates intracellular signaling cascades and promotes transcriptional activation, as well as expression of proinflammatory and antiviral cytokines (Furr and Marriott, 2012). As the principal immune sentinels of the CNS parenchyma, microglia- and astrocyte-mediated immune responses substantially contribute to antiviral immune-mediated events. Recent genomic studies of reactive astrogliosis have identified the neuroinflammatory and neuroprotective activities of astrocytes. Reactive astrocytes are commonly involved in processes of neurodegeneration and neuroinflammation via up-regulation of inflammatory cytokine genes and increased BBB permeability. However, homeostatic astrocytes have been reported to contribute to neuromodulation and neuroprotection via induction of immune tolerance genes (Liddelow and Barres, 2017; Liddelow et al., 2017). Strong pieces of evidence have shown that exposure to PAMPs and microglial-mediated proinflammatory cytokine release contribute to determining astrocyte reactivity (Liddelow et al., 2017). Since microglia are an important component of the intrinsic immune response in the CNS, it is clear that any viral infection will cause direct and indirect microglial responses, which are essential for the anti-viral mechanisms and to a large extent can determine the long-term neurological manifestations of the infection (Vargas et al., 2020). Unlike the innate peripheral cells, the functionality of viral nucleotide-sensing PRR in microglia is not well-known since the BBB is generally thought to prevent peripheral microbes from invading the brain during homeostasis. However, the contribution of microglial nucleotide-sensing mediated antiviral defense to brain pathology in contexts of neuroinvasive viral infections is being appreciated lately (Reinert et al., 2016). Subsequent to viral CNS infection, extracellular nucleotides, such as ATPs are released from neurons, astrocytes or microglia in response to noxious stimuli (Sperlágh and Illes, 2007). The released ATPs are strong chemotactic signals for microglia (Figure 1) and microglia express PRR for their detection (Kawasaki and Kawai, 2014). As infection persists, there is a sustained increase in the levels of these nucleotides, which subsequently trigger the recruitment and phagocytic action of microglia notably via the purinergic receptor P2RY12 signaling pathway (Fekete et al., 2018). This chemotactic tracing contributes to microglial recognition of compromised cells and regulation of phagocytic activity including upon viral infections (Fekete et al., 2018). Furthermore, viruses replicate in cells, accumulating massive amounts of nucleic acids, RNA, and DNA. Cytosolic mitochondrial proteins and dsDNA or ssDNA have been shown to alter microglial and astrocytic activities by triggering intracellular inflammatory pathways (Bajwa et al., 2019). The nucleotide DAMPs accumulate in the cytoplasm when phagocytosed viral particles overwhelm the lysosomal processing pathway. Under cellular stress or DNA damage, dsDNA from the nucleus or mitochondria further infiltrates the cytoplasm of neuronal and glial cells (Roers et al., 2016). DAMPs from neurotropic RNA viruses are prominently recognized by RIG-I dependent mechanisms (Furr et al., 2010; Furr and Marriott, 2012). For instance, RIG-I was markedly upregulated with the concomitant production of IL-6, TNF-α, and antiviral IFN-β when immortalized microglial cells defective in TLR4 or primary astrocyte/microglia were infected with either vesicular stomatitis virus (VSV), 5’ triphosphate double-stranded RNA (50ppp-dsRNA) or 5’-triphosphate single-stranded RNA (50ppp-ssRNA) (Crill et al., 2015). Upon RIG-I knockdown, these effects were significantly attenuated (Crill et al., 2015).

A signaling cascade involving the *stimulator of type I interferon genes* (STING) and *absent in melanoma 2* (AIM2) is an alternative microglial sensing medium for viruses. STING serves as an adaptor molecule upon recognition of a sensor-dsDNA adduct, the best-described being cGAS-dsDNA (Sun et al., 2013; Zhang et al., 2014; Cox et al., 2015). Binding of cGAS-dsDNA to STING forms a complex, which brings in proximity the TANK-binding kinase (TBK1) and its substrate interferon regulatory factor (IRF3) via the recruitment of poly-ubiquitination apparatus. Phosphorylation of the transcription factor IRF3 stimulates the production of type 1 IFN α and β. On the other hand, AIM2 directs the production and secretion of the proinflammatory cytokine IL-1β through its precursor’s proteolytic processing by caspase-1 upon the formation of an active inflammasome complex. Although DNA viruses and retroviruses, via dsDNA, activate this pathway, emerging evidence indicates that positive-stranded RNA viruses can evade immune recognition by suppressing the STING pathway. Positive-stranded RNA viruses seem to interfere with the innate defense mechanisms by disrupting IFN production and its effects (Kabir et al., 2020; Larenas-Linnemann et al., 2020). For instance, the cleavage of STING by non-structural protein (NS) 2B and NS3 proteins was recognized as a conserved strategy used by flavivirus including dengue virus (DENV), WNV, ZIKV, and Japanese encephalitis virus (JEV) to establish infections in human (Aguirre et al., 2012; Yu et al., 2012; Ding et al., 2018). While ZIKV NS1 additionally cleaves cGAS, NS4B of Yellow Fever Virus (YFV) and DENV forms a complex with STING to achieve immune evasion (Ding et al., 2013; Nitta et al., 2013; Chan and Gack, 2016b). The protein product of human coronavirus NL6 and SARS-CoV papain-like proteases particularly disrupt antiviral cGAS-STING-mediated signaling by abolishing ubiquitin-STING conjugation (Sun et al., 2012; Xing et al., 2013; Chen et al., 2014). However, it remains to be investigated whether the inhibition of STING cascade is a significant evasion strategy during SARS-CoV-2 neuroinvasion. Moreover, characterizing the evasion strategies specifically employed by SARS-CoV-2 may present novel pharmacological targets.

Another pathway through which microglia detect CNS viral infections is the classical complement cascade. The complement system, a vital component of the innate immune pathogen defense, consists of approximately 30 proteins and membrane-bound receptors and regulators, which are also involved in pattern recognition and clearance (Ojha et al., 2014; Agrawal et al., 2017). Viruses, such as the WNV and their PAMPs induce complement activation within the CNS (Mehlhop et al., 2005; Vasek et al., 2016). In such infections, the complement system plays a key role in controlling viral propagation. This involves targeting and binding of viral particles and recruitment of proinflammatory peptides and immune cells as well as clearance of cells that express complement receptors. The complement component C3, along with its phagocytic receptor 3 (CR3/CD11b-CD18/Mac-1) expressed on the surface of microglia, recruits immune cells to the site of an injury thereby promoting internalization of synaptic structures. This was demonstrated using mice lacking C3- and its receptor CR3, which had reduced microglial engulfment of synapses (Schafer et al., 2012). Microglia also remove C1q-coated neurites through CR3-mediated internalization (Linnartz et al., 2012). Howbeit, viruses have developed a series of strategies in order to subvert complement detection mechanisms. This includes targeting of recognition molecules and key pathway enzymes, stimulation of proteases that cleave the complement proteins, and/or outright inhibition of the synthesis of complement proteins (Ojha et al., 2014; Agrawal et al., 2017). For instance, several studies report that flaviviruses including WNV, ZIKV, JEV, DENV, and YFV contain a conserved region which codes for a particular complement regulator, non-structural protein 1 (NS-1). This protein is necessary for viral RNA replication (Alcon-Lepoder et al., 2008). As a subversion strategy, the NS-1 protein antagonizes the component C4 (Conde et al., 2016) of the complement cascade, and recruits host major soluble inhibitor of the complement cascade C4 by complexing binding protein and factor H (Kyung et al., 2006). Also, NS-1 inhibits C9 polymerization (Conde et al., 2016), thus aiding flaviviruses to evade the complement detection system and thereby enhancing their survival in host cells.

## Microglial Response During RNA Viral Infections

So far, about 180 species of RNA viruses with the capacity to infect humans have been recognized, and about two new species are added every year on average (Woolhouse et al., 2013). Compared to other groups of viral infection, viral RNA infections are more frightening because they are highly evolving with increased likelihood to infect a new host species due to their outstandingly shorter generation time. The high rate of replication makes the reproductive cycles more error-prone (Holmes, 2010). This gives RNA viruses a potential to quickly produce new strains within a shorter timeframe (Sanjuán and Domingo-Calap, 2016). Although an avalanche of reports have documented neurological symptoms related to COVID-19, the specific role, contributions and implications of microglia during SARS-CoV-2 infection and COVID-19 are still elusive. Thus, proper understanding of the myriads of ways microglia respond to neuroinvasive viral infections may provide insight into how to manipulate the pathogenic mediators to achieve effective viral control thereby advancing therapeutic development.

Antiviral type 1 IFN signaling cascade is pivotal to curtailing viral spread in the CNS parenchyma (Detje et al., 2009). For instance, intranasal VSV instillation at a dose that was harmless to wild-type mice resulted in death within 2–3 days in mice deficient in IFN-α/β receptor (IFNAR). However, the hemizygous mutant of IFNAR, which presented with about 100-fold high viral load, survived 5–6 days before the onset of mortality (Detje et al., 2009). Microglial functions may be pivotal to protecting the brain from neuropathological assaults mediated by viral infection and viral encephalitis (Chen et al., 2019; Hatton and Duncan, 2019). Evidence from rodent studies in the context of viral encephalitis correlated microglial ablation with reduced survival, amplified viral burden, and negative clinical outcomes, including the development of overt neurological diseases and mortalities (Sanchez-Mejias et al., 2016; Fekete et al., 2018; Seitz et al., 2018; Waltl et al., 2018; Sanchez et al., 2019). To unravel the specific contribution of microglial function against neuroinvasive infections, independent investigations in mice have shown that microglial cells can generate antiviral innate immune response (Sorgeloos et al., 2013). Using IFN-β promoter-luciferase reporter mice, Kallfass et al. (2013) observed that astrocytes and F4/80 positive cells, which could be either microglia or infiltrating macrophages, accounted for a significant IFN-β staining in the brain following intraperitoneal La Crosse virus (LACV) infection, even though LACV replicated largely in neurons. However, when mutant LACV-infected mice deficient in NS proteins were used, the IFN-β were mainly detected in astrocytes (Kallfass et al., 2013). Knowing that NS proteins of LACV are an inherent viral strategy to subvert host’s type 1 IFN antiviral responses (Blakqori et al., 2007), this may imply that the mechanism underlining IFN-β production by the F4/80-expressing cells may be non-redundant and more pivotal to the survival of the host cell, particularly when astrocytic antiviral mediators are suppressed. In similitude, the study of Wheeler et al. (2018) observed a significant mRNA expression of IFN-α4, IFN-β, and IL-6, which correlated with viral load in the olfactory bulb and brainstem when mice were intracranially injected with mouse hepatitis virus (MHV), and after microglia were pharmacological depleted with PLX5622 treatment for a week (Wheeler et al., 2018). PLX5622 is an inhibitor of microglia-expressed colony-stimulating factor 1 receptor (CSF1R) required for their survival, although some subsets of microglia are CSF1R negative and thus resistant to this depletion (Erblich et al., 2012). This further emphasizes the presence of extra-microglial antiviral and pro-inflammatory response to various neuroinvasive RNA viral infection, including flaviviruses (Seitz et al., 2018). However, this non-microglial response may be blunt against MHV considering that increasing viral titer and spread co-existed in this context with the expression of antiviral cytokines.

To differentiate the protective role of microglia, treatment of mice with PLX5622, a week before and after an intranasal instillation of neuroattenuated coronavirus MHV infection, was performed, resulting in 100% mortality. However, mice that did not receive PLX5622 survived (Wheeler et al., 2018). Enhanced mortality was also observed in PLX5622-mediated microglia-depleted JEV-infected mice (Seitz et al., 2018). When the timing of PLX5622 treatment was adjusted to day 0 or 3 post infection (p.i.), the survival of mice increased to 10 and 40%, respectively. However, administering PLX5622 on day 6 p.i. did not rescue the MHV-infected mice from mortality (Wheeler et al., 2018). This suggests that microglial activity is pivotal to surviving fatal neurotropic viral infection in mice especially at the early stage of infection. Again, this points to the involvement of microglia in early innate immune responses against neuroinvasive viral infections. A glimpse at the protective mechanism initiated by uninfected microglia against an invading RNA virus in the CNS showed that microglial antiviral type I IFN against VSV was potent at suppressing the anterograde trans-synaptic viral propagation (Drokhlyansky et al., 2017). When eGFP-labeled VSV (VSV-eGFP) or its type 1 IFN-stimulating virus-free interfering particles (DIPs) were injected into the caudate-putamen of mice, FACS-isolated microglial cells (CD11b^+^FCRLS^+^ cells) with or without VSV-eGFP infection upregulated the mRNA expression of *IR7* to a level comparable to microglia from the DIP-injected brain (Drokhlyansky et al., 2017). *IR7*, a master regulator of type 1 IFN, and *Rsad2*, is an IFN stimulated gene (ISG). The DIP injection limited the lateral anterograde spread of the replication-competent rVSV-eGFP across the caudate-putamen circuitry on the contralateral side of the brain. However, microglia infected with VSV-eGFP expressed IFN-β and IL-1β, which were not upregulated in the DIP-injected brain through its antiviral IFN secretion. This suggests that viral-infected microglia are equipped with DIP-equivalent mechanism to limit transneuronal viral transmission and uninfected microglia are inductively primed via IFN-β paracrine signaling emanating from the infected cells. Meanwhile, excessive microglial reaction may result in more damage to the healthy neurons and synapses, hence resulting in further neurodegeneration (Lecours et al., 2018). Whether the additional pro-inflammatory IL-1β production is capable of contributing to neuropathology remains to be investigated.

Following adequate initiation of innate response, microglia may be presenting peptides from the invading neurotropic virus to initiate adaptive immune response and resolution of infection. Using intravital imaging, a recent study by Moseman et al. (2020) demonstrated that intranasal inoculation of mice with VSV and subsequent infection of the CNS was resolved non-cytolytically by cytotoxic T lymphocytes (CTL) through neuronal major histocompatibility complex 1 (MHC-1)-dependent microglial, but not neuronal, presentation of viral peptides. In the study, ablation of neuronal MHC-1 did not affect viral control, whereas depletion of microglia significantly interfered with viral clearance (Moseman et al., 2020). This suggests that lack of microglia may impair T cell recruitment and viral clearance. Following microglial ablation with PLX5622, which resulted in the loss of MHC-II expression in the brain, peripheral CD45^*hi*^CD11b^+^ macrophages with capability to initiate adaptive response were recruited to the brain. This is suggestive of a compensatory mechanism since PLX5622 does not affect their antigen presentation to CD4^+^ or CD8^+^ T cells in response to subclinical intraperitoneal MHV infection in the periphery (Wheeler et al., 2018), although a recent study demonstrated the side effect of PLX5622 on hematopoiesis and peripheral macrophages (Lei et al., 2020). Similarly, brain infiltrating cells expressing markers of microglia and invading monocytes, ionized calcium binding adaptor molecule 1 (IBA1) and Mac-3, were detected following microglial depletion with PLX5622 in a mouse model of picornavirus-mediated viral encephalitis-induced seizure development (Waltl et al., 2018). This emphasizes a microglial role in viral antigen presentation to T cells. Specifically, depletion of microglia with PLX5622 resulted in reduced frequency of CD4^+^ T cells and FOXP3^+^ Tregs in the draining lymph nodes of the brain, diminished IFN-γ expression by virus-specific CD4^+^ T cell response, and enhanced CD8^+^ T cells (Wheeler et al., 2018). However, using a lethal WNV infection, a recent study showed that 2 weeks of PLX5622 pre-treatment in mice was associated with the depletion of both microglia and infiltrating antigen presenting cells within the CNS, which resulted in limited CD8^+^ T cells reactivation and aberrant viral load in the CNS. As opposed to the lethal WNV, the subclinical MHV infection may model the current SARS-CoV-2 infection in human.

With respect to COVID-19, a detailed analysis of 43 post-mortem brains of patients by Matschke et al. (2020) revealed the involvement of microglia in the neuropathology of SARS-CoV-2. The study revealed via an *in silico* analysis of publicly available dataset that both neuronal and non-neuronal cells were vulnerable to SARS-CoV-2 infection with the neurons, oligodendrocytes, microglia, and astrocytes being the most enriched in viral entry apparatus, TMPRSS2/4, ACE2, cathepsin L, and two pore segment channel 2 (TPCN2) expression, respectively. Clinical CNS manifestation of COVID-19 was associated with spike or nucleocapsid mRNA and protein expression in the basal ganglia, cerebellum, frontal cortex, and medulla oblongata as well as pronounced leaky meninges. This is characterized by CTL infiltration clustering around microglia (defined by HLA-DR and CD68 staining) at the brainstem perivasculature (Matschke et al., 2020). Overall, this may imply significant infection and remodeling of perivascular microglia upon SARS-CoV-2 CNS entry and a potential crosstalk with CTL. Previous observations have also confirmed microgliosis in the brainstem (Deigendesch et al., 2020), but the characteristics of the remodeled microglia in response to COVID-19 or SARS-CoV-2 infection remain to be investigated.

## Perspectives: Implications of Microglial Reactivity in SARS-CoV-2 Related Neuropsychiatric Disorders and Neurodegenerative Diseases

It is now generally accepted that many neuropsychiatric disorders and neurodegenerative diseases arise from the influence of environmental factors (Chin-Chan et al., 2015; Khan et al., 2019). The SARS-CoV-2 pandemic may overwhelmingly impact the mental health due to the multiple psychosocial stressors, such as self-isolation/quarantine, fear, anxiety, worry, social restriction, lockdown, and stigmatism created by the disease. Symptoms of neuropsychiatric disorders, such as obsessive-compulsive disorders, insomnia, depression, anxiety and psychoses are being reported in some cases and survivors of SARS-CoV-2 infection (Valdés-Florido et al., 2020). Psychosis is known as one of the neuropsychiatric disorders requiring special care and attention. Of note, since the days of the Spanish Flu pandemic, psychosis of influenza has been documented in many other pandemics (Kêpińska et al., 2020). Anecdotal clinical reports from mental health facilities have recorded increased paranoia amongst persons who are close to infected patients (Brown et al., 2020). Indeed, physical distancing measures have been proposed to serve as risk factor for increased vulnerability to neuropsychiatric disorder including psychosis (Brown et al., 2020). Moreover, evidence has linked both peripheral and neurotropic viral infections with neurodegenerative conditions (Karim et al., 2014). It is now clear that chronic HIV infection correlates with dementia and other neurocognitive disorders (Naghavi, 2018). Babies from ZIKV-infected mothers have microencephaly with consequences on their developmental and cognitive abilities into adulthood, while ZIKV causes neurodegenerative complications including myelitis, neuropathy and Guillain-Barre syndrome (Christian et al., 2019). Of note, different quarters are beginning to sensitize the public about the possibility of post-COVID-19 pandemic of neurodegenerative diseases (Serrano-Castro et al., 2020; Singal et al., 2020), given the neurological symptoms displayed during and after the infection (see section “Evidence of SARS-CoV-2 Neurotropism”). The question begging for answers is how SAR-CoV-2 viruses orchestrate the pathogenesis of neuropsychiatric disorders and neurodegenerative diseases, and whether microglia are involved. Experimental studies have shown that prolonged episode of chronic stress promotes dystrophic microglial phenotype with higher propensity for phagocytosis and apoptosis (Hinwood et al., 2013; Kreisel et al., 2014; Milior et al., 2016; Frank et al., 2019). Ablation of microglial C-X3-C motif chemokine receptor 1 (CX3CR1) in mice resulted in phenotypes associated with autism spectrum disorders including cognitive impairment (Kreisel et al., 2014), social withdrawal (Zhang et al., 2014) and resistance to chronic psychological stress-induced anhedonia- and anxiety-like phenotype (Wohleb et al., 2014; Milior et al., 2016). Also, coronavirus neurovirulence is associated with microglia-mediated up-regulation of proinflammatory signals for the recruitment of blood-derived inflammatory cells (Li et al., 2004; Olajide et al., 2020). Mice infected with mouse hepatitis virus (MHV)-A59 developed a meningoencephalopathy characterized by perivascular inflammation, microglial nodules, and astrocytic proliferation (Lavi et al., 1984; Das Sarma et al., 2000; Li et al., 2004). At 10-day p.i., when viral clearance was achieved in the neurons, viral RNA persisted in the astrocytes and microglia within the olfactory and limbic regions with continued chronic inflammatory demyelination as detected by *in situ* hybridization (Lavi et al., 1984; Das Sarma et al., 2000). Since microglia- and astrocyte-induced neuroinflammation are risk factors for the development of major depressive disorder (Brites and Fernandes, 2015; Troubat et al., 2020), as evident in individuals who committed suicide (Steiner et al., 2008; Schnieder et al., 2014), SARS-CoV-2 neurotropism may trigger or exacerbate neuropsychiatric disorders (Steardo et al., 2020). Also, recent advances in biological psychiatry have suggested that chronic psychosocial stress, such as the one generated by COVID-19 pandemic, could enhance microglial reactivity and impact significantly vulnerability of the brain to various neuropsychiatric disorders including depression, cognitive decline, and schizophrenia (Vargas et al., 2020). This suggests that microglia could contribute significantly to the changes in brain function including altered regulation of neuroendocrine, renin-angiotensin aldosterone tryptophan-kynurenine dysregulation, increased release of proinflammatory cytokines, chemokines, and neurotoxins in stress-sensitive regions (Suzuki et al., 2019; Picard et al., 2021). Moreover, stress-induced microglial remodeling has been linked to increased expression and function of catecholamine reuptake transporters or decrease catecholamine precursors; notably altering the synaptic availability of catecholamine neurotransmitters (Miller et al., 2017) all of which could be associated with the onset of neurological disorders in SAR-CoV-2 patients and/or survivors (Alharthy et al., 2020).

Evidence showed that SARS-CoV-2 infection may result in demyelination suggesting the possibility of immunopathogenic events that lead to the development of neurological disorders including multiple sclerosis (Wu and Perlman, 1999; Khateb et al., 2020). Moreover, an animal model of coronavirus (MHV-4) has been reported to induce demyelination (Fleming et al., 1987). In particular, coronavirus RNA sequences have been observed in the brain and demyelinating structures of multiple sclerosis patients using *in situ* hybridization (Murray et al., 1992). Some of the proposed mechanisms of coronavirus-induced demyelination include cytopathogenic properties of the virus for oligodendrocytes, which is linked to E2 sub-structure (Fleming et al., 1987) and T-cell cross-reactivity (Boucher et al., 2007). This is mainly orchestrated by T cell activation following widespread infection of the CNS parenchyma as lack of MHC I and II in β2-Macroglobulin^–/–^ and A_β_^–/–^ MHV-J2.2-v1 mice or deficiency of CD4 in CD4^–/–^ mice resulted in reduced viral clearance with limited demyelination (Houtman, 1996; Lane et al., 2000). This implies that the recruitment of T cells, which is necessary for viral clearance, may also drive demyelination. Of note, Wu et al. (2000) strongly implicated CD8^+^ T cells, albeit in the presence of rapid viral spread (Marten et al., 2000), in the demyelination of spinal cord that accompanied MHV-JHM-infection in recombination-activating gene 1^–/–^ (RAG1^–/–^) mice lacking T and B cells. A recent study by Kaddatz et al. (2020) demonstrated with a mouse cuprizone induced demyelinating model that CD8^+^ T cells, which predominated around the demyelinating foci, were highly proliferating with extensive cytotoxic granule. This is suggestive of antigenic-primed activated CD8^+^ T cells orchestrated by antigen presenting cells, possibly microglia. Meanwhile, in addition to regulating CD8^+^ T cells during viral neurotropic infection (Waltl et al., 2018), microglia play pivotal roles in the demyelination and remyelination within the CNS (Lampron et al., 2015; Laflamme et al., 2018). Therapeutic treatments of cuprizone-intoxicated mice with BLZ945, a pharmacological CSF1R kinase inhibitor, resulted in striatal and cortical remyelination, which correlated with reduced microglial, but enhanced oligodendroglial density (Beckmann et al., 2018). On the other hand, prophylactic BLZ945 treatment attenuated extensive demyelination in the corpus callosum with the oligodendroglial and microglial dynamics showing similar patterns to the therapeutic treatment (Beckmann et al., 2018). This suggests that microglial reactivity contributes negatively to demyelination. However, in the external capsule—which was not affected by BLZ945 prophylactic treatment—of either cuprizone-treated or triggering receptor expressed on myeloid cells 2 (TREM2) knock-out mice, oligodendrocytes were depleted, with accumulation of myelin debris and axonal damage without any impact on microglial density (Beckmann et al., 2018; Wies Mancini et al., 2019). This may be an indication of dysfunctional microglial phagocytic capacity during demyelination. Indeed, a dynamic gene expression profile with increasing mRNA upregulation of proinflammatory and phagocytic markers, which correlated with peak demyelination and was sustained even after viral clearance, was observed in a non-lethal glial-tropic MHV model of demyelination (Savarin et al., 2018). This may indicate the presence of a continuously reactive microglial phenotype during demyelination with huge implication on long-term demyelination. Nevertheless, cases of SARS-CoV-2-associated inflammatory CNS demyelinating diseases have been documented across the globe. For instance, acute multi-infarct encephalopathy was reported in a 40-year-old woman by Zhang et al. (2020b), while acute transverse myelitis (Durrani et al., 2020; Sarma and Bilello, 2020) and neuromyelitis optica were recently described in patient with SARS-CoV-2 infection (Miskin, 2020). However, it is yet to be unraveled if microglia are the villain in the pathophysiology of the CNS demyelinating diseases associated with SARS-CoV-2 infection.

Although aging has been established as one of the prominent factors responsible for the induction of neurodegenerative diseases, there is now a strong evidence that viral pathogens can precipitate or exacerbate neurodegenerative diseases including Parkinson’s disease (PD) (Matsui and Takahashi, 2009). Existing evidence showed that SARS-CoV-2 infection may worsen PD symptoms. For instance, 10 of 17 PD patients experienced severe PD symptoms and 25 of 214 experienced severe COVID-19 disease in a systematic review of 26 reports Kubota and Kuroda (2021). Also, 148 of 694 (vs. 4,074 of 74,065 in non-PD patients) in a cohort from the United States (Zhang et al., 2020c), as well as 23 of 117 PD patients followed in Spain, the United Kingdom, Iran and Italy across 21 health centers, died of COVID-19 complications (Fasano et al., 2020). Consequently, an area of future concern is whether SARS-CoV-2 infection would expand the exploding cases of PD worldwide (Otero-Losada et al., 2020). In the past, the incidence of parkinsonism was observed to increase after the Spanish flu pandemic in 1918 with people born during the pandemic having 2–3-fold risk to develop parkinsonism compared to people born before to 1888 or after 1924 (Jang et al., 2009; Eldeeb et al., 2020). Since then, association of various viruses and parkinsonism has been reported (Jang et al., 2009; Eldeeb et al., 2020). PD is the fastest growing neurodegenerative disease and movement disorder with a prevalence described to have achieved pandemic status (Dorsey et al., 2018). Among other etiological factors, aging and chronic stress are regarded as a major driver of PD (Reeve et al., 2014; Herrera et al., 2015; Dodiya et al., 2020). Available data confirmed that the severity and transmissibility of SARS-CoV-2 infection is proportional to age and aging remains a major risk factor for SARS-CoV-2 infection and severity of COVID-19 (Nanda et al., 2020). During aging, microglia within the SN take up remodeled dystrophic structural, physiologic and phenotypic features in the healthy state. The microglial “inflammaging” renders SN more vulnerable to any environmental assault which may contribute to the onset or progression of PD (Sharaf et al., 2013; Awogbindin et al., 2020). Moreover, the SN is a brain region enriched with SARS-CoV-2 receptors, including ACE2 and TMPRSS2 (Hamming et al., 2004). Also, a recent article suggested that SARS-CoV-2 may hijack the host by disrupting the mitochondrial, autophagic and lysosomal machineries, which are pivotal to microglial functions including synaptic pruning, neurogenesis, surveillance and phagocytosis (Colonna and Butovsky, 2017; Tay et al., 2017), via direct binding (Gordon et al., 2020). Moreover, infection with Influenza A virus (IAV) and SARS−CoV, which infiltrate the CNS via the olfactory canal like SARS-CoV-2, modulates cellular aging pathways (López-Otín et al., 2013). In addition, the virulent IAV, H1N1, infects dopaminergic neurons resulting in α-synuclein aggregation, the intraneuronal hallmark of PD, via a mechanism related to the inhibition of autophagy (Marreiros et al., 2020). Recently, intracytoplasmic SARS-CoV-2 was detected in the brain of COVID-19 patients but gliosis or microgliosis was not determined (Gomez-Pinedo et al., 2020). This is suggestive of a vacuolation which may template unfolded proteins associated with PD. In the long term, this speculates that SARS-CoV-2, through microglial dysfunction, may have potential to contribute to the progression and onset of PD in the aging population. Viruses, and possibly SARS-CoV-2, can be a precipitating factor in the development of PD. SARS-CoV-2, could be the first “hit” in a two-hit hypothesis, that could sensitize the brain to a later assault. Experimental evidence of a multi-hit PD hypothesis was shown in mice with a synergy between influenza and 1-methyl-4-phenyl-1,2,3,6- tetrahydropyridine (MPTP) toxicity that was eliminated with influenza therapeutics (Sadasivan et al., 2017).

## Concluding Remark

Taken together, microglia could play the role of a double-edged sword during viral neuroinfections. By extension, microglial reactivity, if initiated effectively, could orchestrate the clearance of SARS-CoV-2 in the CNS or trigger neuroinflammation and contribute to the severity of the sequelae associated with SARS-CoV-2 neurotropism. For instance, Olajide et al. (2020) recently demonstrated that SARS-CoV-2 spike S1 elicited a robust NF-kB/NLRP3 inflammasome-mediated pro-inflammatory response in BV-2 microglial cells, although the implication and the receptor(s) mediating the stimulatory effect of S1 glycoprotein were not investigated. Given that the adverse effect of microglial reactivity in the CNS exceptionally outlasts the direct damaging effect of viral neurotropism (Thakur et al., 2021), the present SARS-CoV-2 pandemic provides a global opportunity of proactive research to establish the predicted implications of microglia to avert potential incidence of neuropsychiatric disorders and neurodegenerative diseases, which could be pervasive in the coming years as a result of the growing numbers of cases, survivors and re-current waves.

## Author Contributions

IA and M-ÈT conceived the review manuscript. IA prepared the figure. All the authors contributed to the design, writing, and revision.

## Conflict of Interest

The authors declare that the research was conducted in the absence of any commercial or financial relationships that could be construed as a potential conflict of interest.

## Figures and Tables

**Table:** 

ACE2,	angiotensin-converting enzyme 2
ADEM,	acute disseminated encephalomyelitis
AHLE,	acute hemorrhagic necrotizing encephalitis
AIM2,	absent in melanoma 2
BBB,	blood-brain barrier
cGAS,	cyclic GMP-AMP synthase
COVID-19,	coronavirus disease 2019
CSF,	cerebrospinal fluid
CSF1R,	colony-stimulating factor 1 receptor
CTL,	cytotoxic T lymphocytes
DAMPs,	damage-associated molecular patterns
DENV,	dengue virus
FcR,	Fc receptor
FLAIR,	fluid-attenuated inversion recovery
HCoV,	human coronavirus
hiPSC,	human-induced pluripotent stem cells
hNPCs,	human neural progenitor cells
IFN-α/β,	interferon α/β
IL-1/6,	interleukin 1/6
IRF3,	interferon Regulatory Factor 3
ISG,	Interferon Stimulated Gene
JEV,	Japanese encephalitis virus
LACV,	La crosse virus
MERS-CoV,	middle east respiratory syndrome coronavirus
MHV,	mouse hepatitis virus
MRI,	magnetic resonance imaging
mTOR,	rapamycin
MyD88,	myeloid differentiation primary response 88
NF-κB,	nuclear factor kappa light chain enhancer of activated B cells
NLRs,	nucleotide oligomerization domain (NOD)-like receptors
NOD,	nucleotide-binding oligomerization domain
NRP-1,	neuropilin-1
PAMPs,	pathogen-associated molecular patterns
PRRs,	pattern recognition receptors
RIG-1,	retinoic acid-inducible gene I
RLRs,	retinoic acid-inducible gene (RIG)-I-like receptors
RNA,	ribonucleic acid
SARS-CoV,	severe acute respiratory syndrome coronavirus
STING,	stimulator of type I interferon genes
TBK1,	TANK-binding kinase 1
TLR,	toll-like receptors
TMPRSS2,	rans membrane protease serine 2
TNF-α,	tumor necrosis factor alpha
TRAM,	TRIF-related adaptor molecule
TRIF TIR,	domain-containing adaptor protein-inducing interferon β
WNV,	West Nile virus
YFV,	Yellow fever virus
ZIKV,	Zika virus
